# Tumor Invasiveness, Not Lymphangiogenesis, Is Correlated with Lymph Node Metastasis and Unfavorable Prognosis in Young Breast Cancer Patients (≤35 Years)

**DOI:** 10.1371/journal.pone.0144376

**Published:** 2015-12-11

**Authors:** Zhi-Qiang Zhang, Yu-Zhen Han, Qing Nian, Gang Chen, Shu-Qing Cui, Xing-Yong Wang

**Affiliations:** 1 Ministry of Education Key Laboratory of Child Development and Disorders, Chongqing, China; 2 Key Laboratory of Pediatrics in Chongqing, Chongqing, China; 3 Chongqing International Science and Technology Cooperation Center for Child Development and Disorders, Chongqing, China; 4 Department of Critical Care Medicine, Children’s Hospital of Chongqing Medical University, Chongqing, China; 5 Department of Pathology, Affiliated Hospital of Binzhou Medical University, Binzhou, China; 6 Department of Vascular and Endovascular Surgery, Affiliated Hospital of Binzhou Medical University, Binzhou, China; 7 Department of Nursing care and intervention, Community Health Service Center of North Binzhou, Binzhou, China; University of North Carolina School of Medicine, UNITED STATES

## Abstract

The morbidity rate of breast cancer is on the rise, and the age of onset appears to be trending toward a young age. Breast cancer in young women (BCYW) has a number of distinctive features that differ from breast cancer in middle-aged or elderly women (BCMEW). Lymphatic metastasis plays an important role in the spread of BCYW; however, the mechanisms of lymph node metastasis (LNM) in BCYW are not clear. This study aimed to investigate the mechanism of lymphatic metastasis in BCYW and to evaluate the relationships between lymphangiogenesis, the expression of matrix metalloproteinase 9 (MMP-9) and vascular endothelial growth factor C (VEGF-C) expression, clinicopathological characteristics, and prognosis. Using immunohistochemistry, MMP-9, VEGF-C and the level of lymphatic microvessel density (LMVD) were analyzed in 106 cases of breast invasive ductal carcinoma and 20 cases of breast proliferative lesions. Compared with BCMEW, BCYW had higher MMP-9 expression, higher LNM, and more adverse prognoses. In BCYW, high MMP-9 expression was positively correlated with LNM and impaired survival time. However, in BCMEW, MMP-9 expression was not correlated with LNM or survival time. In addition, high VEGF-C expression was positively correlated with a high level of LMVD in both BCYW and BCMEW. Nevertheless, a high level of LMVD was not correlated with LNM or survival time in the two groups. More importantly, univariate and multivariate survival analysis showed that MMP-9 expression and LNM were independent prognostic factors in BCYW. Our present study indicates that lymphangiogenesis induced by VEGF-C is augmented in breast cancer; however, a higher level of lymphangiogenesis has no significant impact on LNM or survival time. We suggest that tumor invasiveness, rather than lymphangiogenesis, plays an important role in LNM among BCYW. Moreover, MMP-9 and LNM were independent prognostic factors for BCYW.

## Introduction

Breast cancer is one of the most common malignancies and the leading cause of cancer deaths in women worldwide. Tumor metastasis is the primary cause of death for most breast cancer patients. It is well known that the routes of metastasis in breast cancer consist of local invasion, lymphatic metastasis, and hematogenous metastasis. Furthermore, it has been shown that tumor invasion occurs mainly through the lymphatic system in breast cancer; thus, lymphatic metastasis plays a leading role in tumor metastasis. However, the mechanism of lymphatic metastasis in tumors has not yet been fully elucidated [1].

The generally accepted approach for quantifying tumor vascularity has been the quantification of the number of immunohistochemically identified microvessels. However, the study of lymphatic vessel characteristics in malignant tumors has lagged behind the study of blood vessels, largely due to the lack of availability of lymphatic-specific markers. In recent years, these types of specific markers, such as LYVE-1, Prox-1, and D2-40, have been characterized and have become commercially available. Consequently, the study of the characteristics of lymphatic vessels in malignant tumors has recently become feasible [2]. It has been reported that tumor cells can induce the formation of new lymphatic vessels in a process known as lymphangiogenesis [3]. Lymphatic microvessel density (LMVD), which is the calculation of positively stained lymphatic vessels per tumor area, has been used to assess lymphangiogenesis in tumor specimens. Newly formed lymphatic vessels are composed of a single layer of endothelial cells, and the junctions between these endothelial cells are not tight. Consequently, tumor cells can easily invade the lymphatic vessels and metastasize to lymph nodes or distant sites. These findings suggest that lymphatic vessel characteristics may be an applicable indicator to predict the metastatic ability of breast tumor cells.

Women younger than 35 years old are less likely to develop breast cancer according to clinical data. However, in recent years, the morbidity rate of breast cancer has risen, and the age of onset appears to be trending toward a younger age [4]. Moreover, the majority of previous studies has shown that BCYW has a relatively high degree of malignancy and an unfavorable prognosis [5–8]. Similarly, our previous work also showed that BCYW has a higher rate of LNM and a poorer prognosis. It has been reported that breast tumor cell intravasation into lymphatic vessels is a critical step in lymphatic metastasis; therefore, it is imperative to elucidate the mechanism of lymphatic metastasis and to clarify the regulatory factors of lymphatic metastasis in breast cancer. However, few studies have focused on the relationships between lymphangiogenesis, lymphatic metastasis, molecular markers, and prognosis in human breast cancers, especially in BCYW.

VEGF-C is the most important lymphangiogenic factor produced by tumor and stromal cells [9]. Several investigations have demonstrated that lymphangiogenesis is correlated with VEGF-C expression [10]. It has been reported that VEGF-C can promote lymphangiogenesis and lymphatic metastasis of tumors, activating the signaling pathway for lymphangiogenesis by merging with VEGFR-3 on the surface of lymphatic endothelial cells [11]. MMP-9, which belongs to the gelatinase subfamily of MMPs, has the unique ability to degrade Type IV collagen (the major component of the basement membrane) and other essential extracellular matrix components. It has been demonstrated that degradation of the extracellular matrix is an essential step in the formation of tumor metastasis. Wu and colleagues found that MMP-9 expression was associated with LNM and suggested that MMP-9 may take part in the early progression of lymphangiogenesis and lymphatic metastasis in breast cancer [12]. Moreover, it has been reported that elevated expression of MMP-9 is positively associated with tumor invasion, metastasis, and poor prognosis in breast cancer [13]. In addition, MMP-9 exerts diverse roles in the tumor dissemination process, such as tumor invasion, tumor-induced angiogenesis, and immunomodulation of the tumor microenvironment [14]. These findings indicate that the combined evaluation of of MMP-9 and VEGF-C expression and lymphatic vessel characteristics is useful in predicting the probability of malignant metastasis and can also provide theoretical guidance for the clinical treatment of breast cancer patients.

To evaluate lymphatic vessel characteristics in BCYW, we calculated the LMVD and investigated the relationship between LMVD and the expression of VEGF-C and MMP-9 by immunohistochemical staining. Furthermore, to ascertain the characteristics of lymphatic metastasis in BCYW, we investigated the relationship between lymph node status and LMVD and the expression of VEGF-C and MMP-9 expressions in clinical samples. Notably, to gain a deeper understanding of the features of BCYW, we further explored the clinicopathological features, survival rate, LMVD, and expression of VEGF-C and MMP-9 in BCYW as well as BCMEW and drew comparisons between these two groups. To our knowledge, this is the first study to investigate the lymphatic vessel characteristics and the expression of MMP-9 and VEGF-C in clinical samples of BCYW (< 35 years).

## Materials and Methods

### 2.1. Patients and tumor samples

All tissues used for the study were retrieved from the archives of the Pathology Department at the Affiliated Hospital of Binzhou Medical University from 2002 to 2009. The study was approved by the local ethics committee. Approximately 106 female breast cancer patients pathologically diagnosed with invasive ductal carcinoma were included in the study group. Of the 106 patients, 51 were 35 years old or younger (median age 34.0 years, range 26–35 years) [15], and 55 were randomly selected in the archives from those who were older than 40 years old (median age 50.0 years, range 40–67 years). All the patients in the study group had undergone modified radical operation and had not received any radiotherapy, chemotherapy, or immunotherapy before the operation. The 20 patients with breast proliferative lesions in the control group (median age 35.0 years, range 26–45 years) were randomly selected from the archives during the same period from 2002 to 2009. The follow-up period was completed in May 2015 with a 3–6 month interval. The median follow-up duration was 94.5 months (range 12–120 months). Disease-free survival (DFS) was defined as the duration from diagnosis to any tumor-related relapse or death due to breast. Overall survival (OS) was defined as the duration from diagnosis to the date of death from any cause or the date of last follow-up.

### 2.2 Ethical approval

This study was approved by the Ethics Committee of the Affiliated Hospital of Binzhou Medical University and was performed in accordance with the Helsinki declaration on the use of human subjects in research. The patients or their guardians signed informed consent and agreed to the use of their medical records and samples in research before participating in this study. To protect the private information of the patients enrolled in the present study, the patients were coded with serial numbers, and their private data were removed once the analysis was completed.

### 2.3 Immunohistochemistry

Serial section slides (5 microns) were obtained from paraffin-embedded specimens, and the paraffin medium was removed. The slides were then re-hydrated by passing them through descending serial alcohol dilutions. After antigen retrieval, slides were incubated in antibodies to estrogen receptor (ER), progesterone receptor (PR), human epidermal growth factor receptor 2 (HER2), VEGF-C, MMP-9 (all from Zhongshan Golden Bridge Biotechnology Co., Ltd.), and LYVE1 (Abcam) overnight at 4°C. After washing, slides were incubated in secondary antibody for 30 min at room temperature, washed three times, visualized using DAB, rinsed in distilled water and counterstained with hematoxylin. Finally, the slides were mounted and cover-slipped with neutral balsam. Slides stained with PBS instead of primary antibody were used as negative controls. Slides known to stain positively were used as positive controls.

### 2.4 Microscopic examination

Immunostaining was independently examined by two clinical pathologists who had no knowledge of the clinical data. LYVE1 was considered positive when intense and diffuse staining was seen along the endothelium. LMVD was determined from the counts of LYVE1-positive vessels. The immunostained sections were first scanned at a low magnification, and areas with the highest positively stained vessel density (termed “hot spots”) were identified. The number of positively stained lymphatic vessels in three high-power fields (200×) in the selected areas was counted. LMVD was determined as the mean value of vessel counts. VEGF-C-, MMP-9-, and HER2-positive cells showed brown-yellow particles in their cytoplasm. ER- and PR-positive cells showed brown-yellow particles in their nuclei. Three high-power fields (200×) were randomly selected for each sample. Staining intensity and the percentage of positive tumor cells were assessed according to the methods described by Hao [16]. Intensity was scored as 0 (negative), 1 (weakly positive), 2 (moderately positive) and 3 (strongly positive). The fraction of positive-staining tumor cells was categorized as follows: 0 (<5% positive cells), 1 (6–25% positive cells), 2 (26–50% positive cells), 3 (51–75% positive cells) and 4 (>75% positive cells). The individual labeling scores were generated by multiplying the intensity and the percentage scores: 0 (negative), + (1–4), ++ (5–8), and +++ (9–12). Among these, 0 and + samples were determined to represent low expression; otherwise, they were designated as representing high expression. Molecular subtype classification defined as follows: Luminal A (ER-positive and/or PR-positive and HER2-negative); Luminal B (ER-positive and/or PR-positive and HER2-positive); HER2-positive (ER-negative and PR-negative and HER2-positive); Triple-negative (ER-negative and PR-negative and HER2-negative).

### 2.5 Statistical analysis

All statistical analyses were performed with SPSS for Windows version 13.0 (SPSS Inc., Chicago, IL, USA). Pearson’s chi-square test or the Kruskal-Wallis H test was used to compare the clinicopathological parameters in BCYW and BCMEW and to compare the expression of VEGF-C and MMP-9 in BCYW and BCMEW. LMVD were expressed as the means±SD, medians, or interquartile ranges according to the distribution features of the LMVD data. The Mann-Whitney *U* test was adopted to assess LMVD in BCYW and BCMEW. The correlations among the expression of VEGF-C, MMP-9, levels of LMVD, and lymph node status were calculated by the chi-square test and Spearman’s correlation coefficient. For survival analysis, the end points were the incidence of DFS and OS. Kaplan-Meier survival curves were employed to evaluate DFS and OS, and the differences in the survival curves were assessed by the log-rank test. Univariate and multivariate analysis using the Cox proportional hazards model were performed to evaluate the independent predictive effect of the covariates. All statistical tests were two-sided and differences were considered to be statistically significant when P < 0.05.

## Results

### 3.1 Comparisons between clinicopathological parameters in BCYW and BCMEW

Clinicopathological parameters in BCYW and BCMEW are shown in Table 1. The difference in LNM status between BCYW and BCMEW was statistically significant. Data analysis indicated that the rate of LNM was higher in BCYW compared to BCMEW. There were no significant differences in tumor size, histological grade, molecular subtype, and TNM staging between BCYW and BCMEW group (all P >0.05). Although the ER- and PR-positive expression rates were higher in BCMEW and HER2-positive expression rate was higher in BCYW, these values did not reach statistical significance.

**Table 1:** clinicopathological parameters and VEGF-C, MMP-9 expression in BCYW and BCMEW group.

	Case No.	BCYW	BCMEW	P
Tumor size				
≤2 cm	70	35	35	
>2 cm	36	16	20	0.588
Histological grade				
I	14	4	10	
II	56	30	26	
III	36	17	19	0.247
ER				
Negative	52	29	23	
Positive	54	22	32	0.122
PR				
Negative	48	27	21	
Positive	58	24	34	0.127
HER2				
Negative	57	23	34	
Positive	49	28	21	0.085
Molecular subtype				
Luminal A	44	18	26	
Luminal B	19	9	10	
HER2 positive	30	19	11	
Triple negative	13	5	8	0.242
LNM				
no	62	24	38	
yes	44	27	17	0.021
TNM staging				
I	46	22	24	
II	33	13	20	
III	27	16	11	0.312
VEGF-C				
Negative	42	22	20	
Positive	64	29	35	0.476
MMP-9				
Negative	61	24	37	
Positive	45	27	18	0.035

### 3.2 LMVD, VEGF-C, and MMP-9 expression in breast cancers and breast proliferative lesions

**Fig 1:**
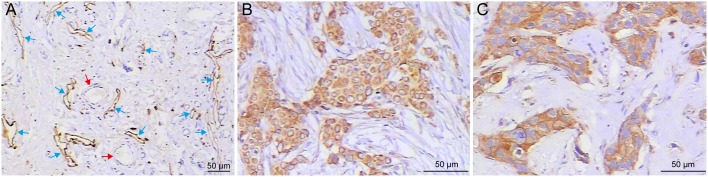
Representative images of LYVE1-positive lymphatic vessels and immunohistochemical staining of VEGF-C and MMP-9. (A) The hot spots in LYVE1 immuno-stained sections. LYVE1-positive lymphatic vessels were thin-walled structures with irregular lumens, mainly present at the tumor periphery (blue arrows). Blood vessel endothelium near LYVE-1-positive lymph vessels were negatively stained (red arrows). (B) Positive expression of VEGF-C. (C) Positive expression of MMP-9. Diffuse and strong positive VEGF-C and MMP-9 immunostaining was mainly observed in the cytoplasm of breast cancer cells.

Positive staining of LYVE1 was seen in the cytoplasm of endothelial cells (Fig 1A). LYVE1-positive vessels were thin-walled structures with irregular lumens devoid of RBC, and they were composed of a single layer of endothelium surrounded by razor-thin connective tissue without smooth muscle fibers. Lymphatic vessels mainly exist in the peripheral area of the tumor and are rarely seen inside the tumor mass (S1 Fig). LYVE-1-positive lymph vascular structures in the tumor peripheries were flattened and cluttered compared to those of the control (S1 Fig). This type of vessel was dispersively located in the interlobular stroma, adipose tissue, and areas close to blood vessels. Based on the features mentioned above, these vessels were identified as lymphatic vessels. The endothelial cells of the blood vessels near the lymphatic vessels were not stained, and RBCs could be found in the blood vessel cavity. Both VEGF-C- and MMP-9-positive staining were found in the cytoplasm of tumor cells, in which brown-yellow particles were dispersively located (Fig 1B and 1C).

### 3.3 Comparisons of LMVD in BCYW and BCMEW

**Fig 2:**
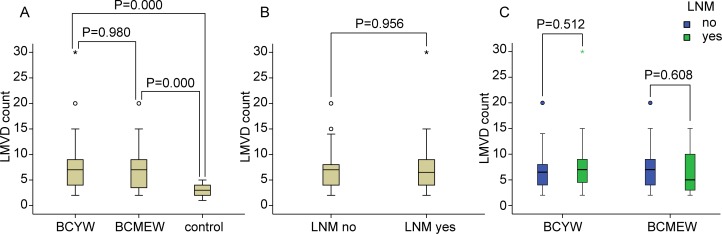
Comparisons of LMVD in different subgroups. (A) LMVD in the BCYW and BCMEW groups and in the control group. LMVD in either BCYW or BCMEW was significantly higher than that in the breast proliferative lesions. However, no significant difference was found between the BCYW and BCMEW groups. (B) LMVD in breast cancer patients with or without LNM was not significantly different. (C) LMVD was not significantly different according to lymph node status in both the BCYW and BCMEW groups (Mann-Whitney *U*-test).

LMVD levels in the breast cancer and control groups are shown in Fig 2. The difference in LMVD between BCYW or BCMEW and the control group was statistically significant, while the difference in LMVD between the BCYW and BCMEW groups was not statistically significant (Fig 2A). Statistical analysis indicated that LMVD in both the BCYW and BCMEW groups was higher than that in the control group. There was no significant difference in LMVD between breast cancer groups with or without LNM (Fig 2B). In both the BCYW and BCMEW groups, there were no significant differences in LMVD according to lymph node status (Fig 2C). Moreover, there was no significant difference for LMVD according to tumor size, histological grade, or TNM stage (data not shown).

### 3.4 Comparisons of VEGF-C and MMP-9 expression between BCYW and BCMEW

The differences in VEGF-C or MMP-9 expression between the BCYW and control groups and between the BCMEW and control groups were statistically significant (P = 0.000). The difference in MMP-9 expression between the BCYW and BCMEW groups was statistically significant, while the difference in VEGF-C expression between the two breast cancer groups was not statistically significant (Table 1). Statistical analysis indicated that the positive expression rate of both VEGF-C and MMP-9 in the BCYW and BCMEW groups was higher than that in the control group. Furthermore, the positive expression rate of MMP-9 in the BCYW group was higher than that in the BCMEW group.

### 3.5 Associations between VEGF-C or MMP-9 expression and LMVD in BCYW and BCMEW

According to the frequency distribution of LMVD and literature reports, samples were designated “low LMVD” when the LMVD count was lower than or equal to 5; otherwise, they were designated “high LMVD.” The associations between VEGF-C expression and LMVD and between MMP-9 expression and LMVD were analyzed by chi-square test (Table 2). The association coefficient of LMVD and VEGF-C in the BCYW and BCMEW groups revealed that VEGF-C expression was positively associated with LMVD. However, the association coefficient of LMVD and MMP-9 in the two groups revealed that MMP-9 expression was not significantly associated with LMVD.

**Table 2:** Associations between VEGF-C, MMP-9 expression and LMVD in BCYW and BCMEW group.

		VEGF-C				MMP-9			
	n	negative	positive	P	*r*	negative	positive	P	*r*
BCYW									
LMVD low	21	14	7			10	11		
LMVD high	30	8	22	0.005	0.369	14	16	0.947	0.009
BCMEW									
LMVD low	23	13	10			16	7		
LMVD high	32	7	25	0.008	0.335	21	11	0.759	0.041

### 3.6 Associations between LNM and LMVD, VEGF-C, or MMP-9 expression in BCYW and BCMEW

The association between LMVD, VEGF-C, or MMP-9 expression and LNM was analyzed by chi-square test (Table 3). The association coefficient of LMVD, VEGF-C and LNM in the BCYW and BCMEW groups revealed that LMVD or VEGF-C was not significantly associated with LNM. In addition, the association coefficient of MMP-9 and LNM revealed that MMP-9 was not significantly associated with LNM in the BCMEW group. However, the statistical analysis showed that MMP-9 was significantly associated with LNM in the BCYW group.

**Table 3:** Association between LMVD or MMP-9 expression and lymph node status in BCYW and BCMEW group.

		LMVD				VEGF-C				MMP-9			
	n	low	high	P	*r*	low	high	P	*r*	low	high	P	*r*
BCYW													
LNM no	24	11	13			11	13			17	7		
LNM yes	27	10	17	0.524	0.089	11	16	0.714	0.051	7	20	0.001	0.410
BCMEW													
LNM no	38	14	24			14	24			25	13		
LNM yes	17	9	8	0.263	0.149	6	11	0.912	0.015	12	5	0.726	0.047

### 3.7 Associations between MMP-9 expression and clinicopathological parameters in BCYW and BCMEW

In the BCYW group, MMP-9 expression was significantly associated with tumor size, TNM staging, ER and PR status (Table A in S1 File). In the BCMEW group, although it appeared that MMP-9 expression was associated with PR status, it did not reach statistical significance. However, there was no significant correlation between MMP-9 expression and histological grade in the BCYW or BCMEW groups. Moreover, there was no significant correlation between MMP-9 expression and tumor size, TNM staging, ER status, HER2 status, or molecular subtype in the BCMEW group. Although it appeared that MMP-9 expression was associated with HER2 status and molecular subtype in the BCYW group, the analysis did not show statistical significance.

### 3.8 Survival analysis

**Fig 3:**
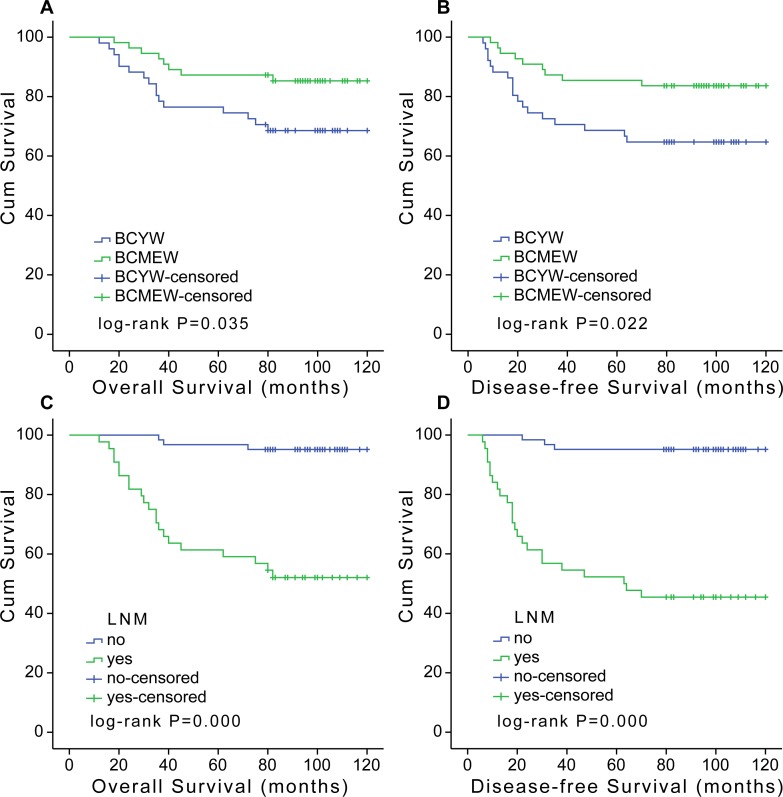
Kaplan-Meier OS (A and C) and DFS (B and D) curves. (A) BCYW (age **≤**35) had a significantly worse OS compared with BCMEW (age >40). (B) BCYW had a significantly worse DFS compared with BCMEW. (C) Breast cancer patients with LNM had a significantly worse OS compared to those without LNM. (D) Breast cancer patients with LNM had a significantly worse DFS compared to those without LNM.

At a median follow-up of 94.5 months, 27 RFS events and 24 OS events were registered. Kaplan-Meier analysis was used to evaluate the DFS and OS of patients with breast cancer. The BCYW group had a lower OS and DFS rate compared with the BCMEW group (Fig 3A and 3B). Fig 3C and 3D showed the Kaplan-Meier curves of OS and DFS for breast cancer groups with or without LNM. The results showed that patients with LNM had a worse OS and DFS than those without LNM.

Significant predictors for the death of the BCYW are summarized in Tables B and C in S1 File. Univariate and multivariate analyses were performed to identify factors associated with OS and DFS. In BCYW, univariate analysis identified tumor size, LNM, TNM stage, and MMP-9 expression levels to be adverse prognostic factors for OS and DFS (P<0.05 for each factor). Univariate analysis identified triple-negative subtype to be adverse prognostic factors for OS (P = 0.046). Although there was a trend for triple-negative subtype serving as an adverse prognostic factor for DFS, the analysis did not show statistical significance (P = 0.182). Multivariate analysis indicated that high MMP-9 expression was an independent factor for OS (RR, 5.354; 95%CI, 1.171 to 24.481; P = 0.004) or DFS (RR, 6.466; 95%CI, 1.443 to 28.968; P = 0.015) in BCYW, as well as LNM for OS (RR, 5.282; 95%CI, 1.156 to 24.132; P = 0.032) or DFS (RR, 6.466; 95%CI, 1.443 to 28.968; P = 0.015).

In BCMEW, univariate analysis identified histological grade, HER2, and LNM as adverse prognostic factors for OS and DFS. Although there was a clear trend for ER status serving as a favorable prognostic factor for OS by univariate analysis in BCMEW, the analysis did not show statistical significance (P = 0.064). Nevertheless, univariate analysis showed that ER status was a favorable prognostic factor for DFS by univariate analysis in BCMEW (P = 0.034). Multivariate analysis also indicated that histological grade and LNM were significant predictors for both OS and DFS (histological grade, P = 0.013 and 0.014; LNM, P = 0.009 and 0.004, respectively). However, no significant correlations were observed between LMVD or VEGF-C and any survival outcome.

## Discussion

Previous reports indicated that breast cancer is a relatively rare disease in young women, although earlier studies have also shown that women **≤**35 years have distinctly worse survival than middle-aged women [6]. There is still no unanimous definition for “young age breast cancer” or “young breast cancer.” Multicenter clinical studies considered 35 years to be the age boundary [15]. Han and colleagues analyzed data on 9,885 breast cancer patients aged <50 years, and their team found that age less than 35 years was a reasonable cut-off for defining young age-onset breast cancer [15]. In our present study, we also used 35 years as the age boundary.

There are a number of different clinicopathological features between BCYW and BCMEW. Anders and colleagues showed that younger women demonstrated a larger tumor size, higher grade tumors, and greater incidence of lymph node positivity in their study [17]. A number of findings have agreed with Anders’ work [5, 18]. Wei’s study included a total of 1913 cases of primary breast cancer, with 283 cases diagnosed as young patients [19]. Their data analysis showed that no significant difference was observed in tumor size, TNM staging, or LNM between BCYW and BCMEW. The results from a number of other studies are in accordance with Wei’s findings [7, 20]. In the present study, we found that the differences in tumor size and histological grade between BCYW and BCMEW were not statistically significant, which was in agreement with Wei’s findings. Nevertheless, there was a trend towards lower positive rates of ER or PR in BCYW than in BCMEW in our study. In addition, there seemed to be a trend that the positive rate of HER2 was higher in BCYW than in BCMEW. These discrepancies could be due to several reasons. First, the patients studied were from different countries and regions. The inconsistencies in patients’ races and nationalities may have had a noticeable impact on the results; for example, the morbidity rate of young breast cancer patients (**≤**35 years old) was less than 4% in Western countries, but the proportion of patients in this age range was reported to be 10–15% in China [19]. Second, the disparities in economic and social status among patients may also have impacted the results. Lastly, random error may have influenced the results. These findings indicate that the BCYW has distinct features from BCMEW. Consequently, we should focus on the unique characteristics of the young populations to clarify the pathogenic mechanisms of BCYW.

It was reported that the greater the number of metastatic lymph nodes, the worse the prognosis. Hutter and colleagues found that the number of LNM was one of the decisive factors affecting the prognosis of breast cancer [21]. Our findings showed that the BCYW group had a higher LNM rate and a more adverse prognosis than the BCMEW group, which was in accordance with other studies [22]. Patients with LNM have a more unfavorable prognosis than those without LNM. Therefore, it is necessary to elucidate the mechanism underlying LNM in young populations. However, whether lymphangiogenesis plays an important role in LNM of BCYW has yet to be evaluated. Solid evidence for a relationship between lymphangiogenesis and LNM or prognosis in human breast cancer is still lacking. Elucidation of the mechanism underlying LNM could have implications for the clinical management of BCYW.

The metastatic spread of tumor cells is the main cause of death in breast cancer patients. Metastasis involves complex processes, such as the degradation of extracellular matrix, intravasation into lymphatic and/or blood vessels, clonogenic growth in secondary sites and neoangiogenesis [16]. The routes of tumor metastasis mainly include local invasion, lymphatic metastasis, and circulatory metastasis. In breast cancer, lymphatic metastasis is considered to be a key process among the three routes. Lymphatic metastasis may depend on the capacity of tumor cells to induce lymphangiogenesis and to invade the lymphatic system. It is not clear whether lymphatic metastasis is achieved via the invasion of newly formed lymphatic vessels and/or by the invasion of preexisting lymphatic vessels [23].

The study of lymphatic vessel characteristics in malignant tumors has lagged behind that of blood vessels largely due to the absence of specific markers for lymphatics and the lack of detailed knowledge concerning the molecular mechanisms of lymphangiogenesis. In recent years, the type of specific markers that are specifically expressed and localized in lymphatic endothelial cells, such as LYVE-1, vascular endothelial growth factor receptor-3, D2-40, Prox-1, and podoplanin have been identified and become commercially available. The discovery of these lymphatic endothelial cell markers has enabled the distinction between lymphatic and blood vessels at the capillary level, resulting in marked advances in the study of lymphangiogenesis. LYVE1, a type of hyaluronan receptor, is one of the most specific lymphatic endothelial markers. In this study, we used LYVE1 as the specific lymphatic markers and found that LYVE1 was expressed specifically in the lymphatic endothelial cells in breast cancer tissues without being expressed in blood vessel endothelial cells. Therefore, LYVE1 could serve as a reliable lymphatic marker for the study of lymphatic metastasis in breast cancer.

In our study, LYVE1 immunostaining revealed that lymph vessels were mainly present in the peritumoral lesions of breast tumors and were rarely observed inside the tumor. This may be due to the high hydrostatic pressure in the intratumoral lesions, which is not conducive to the formation of lymphatic vessels.It may also be because the immunophenotype of newly formed lymphatic vessels is different from typical lymphatic vessels distributed in the normal structure. A plausible hypothesis is that tumor tissues might use a different lymphatic drainage mechanism, such as “lymphogenic mimicry,” similar to the vasculogenic mimicry described in other studies [24, 25]. The analysis of our data on LMVD showed that lymphatic vessels in the tumor periphery significantly exceeded those of the control proliferative lesion, which indicated that lymphatic vessels in the tumor periphery are mainly induced by tumor cells in lymphangiogenesis. Furthermore, data analysis showed that VEGF-C expression in tumor cells significantly exceeded the expression of control proliferative lesions, which further supported the explanation mentioned above.

In this study, we found that LMVD in both the BCYW and BCMEW groups was higher than that in the proliferative lesion group. However, LMVD was not significantly different between the BCYW and BCMEW groups. Furthermore, our statistical analysis showed that LMVD was not associated with LNM. In addition, LMVD was not associated with tumor size or with histological grade in both the BCYW and BCMEW groups. These findings are in line with the results of Williams et al. [26, 27]. According to our findings, we conclude that the number of lymphatic vessels around the tumor mass in breast cancer may not play a major role in the process of LNM, and lymphangiogenesis might be not be involved in LNM. Initially, we proposed that BCYW would exhibit a higher degree of lymphangiogenesis than BCMEW. Correspondingly, we also proposed that a higher degree of lymphangiogenesis in BCYW would be accompanied by an increased risk of LNM. Interestingly, in our study, we found that the degree of lymphangiogenesis showed no significant relationship with LNM in both BCYW and BCMEW populations. Furthermore, we found that the degree of lymphangiogenesis showed no significant relationship with DFS or OS. However, in the present study, we found that the overexpression of MMP-9 was significantly associated with a higher rate of LNM in BCYW. Based on the present data and on those available in the literature, we propose that lymphangiogenesis induced by the tumor may not play an important role in the complex processes of lymphatic metastasis.

Indeed, newly formed lymphatic vessels in the tumor periphery offer an opportunity for tumor cells to invade and migrate to the lymphatic draining system. However, in BCYW, the induced newly formed lymphatic vessels may be dysfunctional and exhibit difficulty in connecting to the lymphatic drainage system. Moreover, the newly formed lymphatic vessels, which lack connective tissue support, tend to be occluded by the compression of tumor tissue. Thus, lymphangiogenesis in the tumor periphery does not have a significant relationship with LNM. It has been reported that young breast tumors have more aggressive biological features, and age is an independent factor for prognosis in several studies. Therefore, tumor cells in the young breast cancer population could exhibit a more aggressive method of growth to more easily detach from the tumor colony, spread to the surrounding normal tissues, and invade the pre-existing or newly formed functional lymphatic vessels.

VEGF-C, an important member of the VEGF family, has been demonstrated to participate in the regulation of lymphangiogenesis by activating its special receptor, vascular endothelial growth factor receptor-3, on the surface of lymphatic endothelial cells [28, 29]. In our study, we showed that the positive expression rate of VEGF-C in both the BCYW and BCMEW groups was higher than that in the control group, which was in accordance with previous reports [30, 31]. It has been reported that elevated VEGF-C expression is associated with higher lymph vessel density and LNM in breast cancer [23, 32]. Timoshenko and colleagues reported that the over-expression of VEGF-C in the tumor micro-environment was associated with LNM and a poor prognosis in breast cancer patients [33]. In our research, we also showed that the higher expression of VEGF-C was significantly associated with increased LMVD. However, our present results indicate that higher expression of VEGF-C is not associated with LNM or poor prognosis in breast cancer patients.

MMP-9 can disintegrate and destroy the collagen and fibronectin of the extracellular matrix. The overexpression of MMP-9 in the tumor tissue can facilitate the tumor cells to penetrate through the basilar membrane and infiltrate the adjacent tissues, resulting in tumor metastasis [34]. Previous findings indicated that the overexpression of MMP-9 in breast cancer tissues was significantly associated with LNM [16, 35]. Our data analysis showed that the expression of MMP-9 in either BCYW or BCMEW group was higher than that in the control group, which was in line with literature. In addition, the overexpression of MMP-9 in both the BCYW and BCMEW groups was significantly associated with LNM. These findings indicate that MMP-9 expression is increased in breast cancer, which suggests that MMP-9 may play an important role in LNM in BCYW.

Based on these findings, we suggest that breast tumor cells that express high levels of MMP-9 may be more invasive and have a greater probability of passing through the extracellular matrix and entering the lymphatic system. Moreover, we found that the positive expression rate of MMP-9 in BCYW was significantly higher than that in the BCMEW, which indicates that disease in BCYW tends to be more aggressive than in BCMEW. Furthermore, we demonstrated that high MMP-9 expression was an independent prognostic factor for BCYW. In addition, we found that positive MMP-9 expression was associated with larger tumor size, higher TNM staging, negative ER and PR in the BCYW. We also found that positive MMP-9 expression tended to be associated with positive HER2 in the BCYW and negative PR in the BCMEW. A number of findings have indicated that HER2-positive breast cancers are associated with higher local recurrence rates and poorer OS than ER- and PR-positive breast cancers [36, 37]. Therefore, we propose that MMP-9 might be an effective target for therapeutic strategies to prevent or retard LNM in BCYW. Inhibiting the expression or blocking the function of MMP-9 to reduce tumor invasiveness may be an effective therapeutic strategy for the treatment and management of BCYW.

In summary, for the first time, we used the reliable lymphatic endothelial marker LYVE1 to estimate LMVD and assessed VEGF-C and MMP-9 expression by immunohistochemical staining in BCYW. The LMVD assays and the assessment of VEGF-C and MMP-9 expression demonstrated relationships between lymphangiogenesis and tumor invasiveness with tumor clinicopathological parameters. We found that BCYW were able to develop LNM and had an unfavorable prognosis. Furthermore, this study indicates that MMP-9 is an important factors facilitating the spread of tumor cells through the lymphatic system. Moreover, our present study demonstrates that tumor invasiveness, not lymphangiogenesis, plays an import role in LNM among BCYW. LNM in BCYW may be caused mainly by increased tumor invasiveness rather than lymphangiogenesis. Further studies are required to fully elucidate the detailed mechanisms of LNM in young breast cancer. Moreover, our findings suggest that the targeted inhibition of MMP-9 may be an effective adjuvant therapy for BCYW. Furthermore, our findings could provide guidance for the clinical treatment of cancer, and the targeted inhibition of tumor lymphangiogenesis may not be an effective therapeutic method for young breast cancer patients.

## Supporting Information

S1 Fig Representative images of LYVE1 positive lymphatic vessels in breast cancer and control tissues. (A) Lymphatic vessels mainly exist in the periphery area of the tumor and are rarely seen inside the tumor mass. (B) Lymphatic vessels in the contol group.
(TIF)

S1 File Associations between MMP-9 expression and clinicopathological parameters in BCYW and BCMEW (Table A). Univariate and multivariate analysis of overall survival in BCYW and BCMEW (Table B). Univariate and multivariate analysis of disease-free survival in BCYW and BCMEW (Table C). (DOCX)
