# Supplementary figures and images for: Tumor Invasiveness, Not Lymphangiogenesis, Is Correlated with Lymph Node Metastasis and Unfavorable Prognosis in Young Breast Cancer Patients (≤35 Years)
Source: PLoS One. 2015 Dec 11;10(12):e0144376. doi: 10.1371/journal.pone.0144376 (PMC4676633; doi:10.1371/journal.pone.0144376)

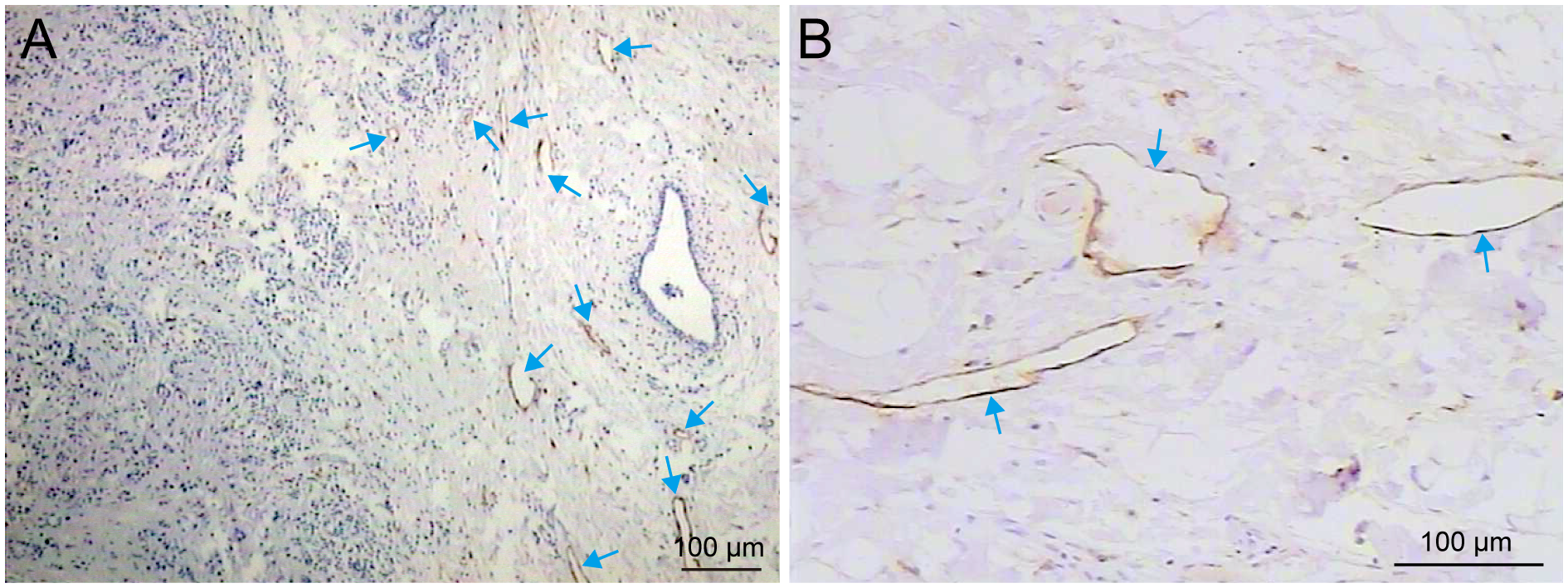

Supplement: S1 Fig — (A) Lymphatic vessels mainly exist in the periphery area of the tumor and are rarely seen inside the tumor mass. (B) Lymphatic vessels in the contol group. (TIF) [file pone.0144376.s001.tif]
