# Supplementary material for: Tumor Invasiveness, Not Lymphangiogenesis, Is Correlated with Lymph Node Metastasis and Unfavorable Prognosis in Young Breast Cancer Patients (≤35 Years)
Source: PLoS One. 2015 Dec 11;10(12):e0144376. doi: 10.1371/journal.pone.0144376 (PMC4676633; doi:10.1371/journal.pone.0144376)
Supplement: S1 File — (DOCX) [file pone.0144376.s002.docx]

**Table A Associations between MMP-9 expression and clinicopathological parameters in BCYW and BCMEW**

|  |  | BCYW | | | |  |  | BCMEW | | | |
| --- | --- | --- | --- | --- | --- | --- | --- | --- | --- | --- | --- |
|  | n | MMP-9 negative | MMP-9 positive | P | *r* |  | n | MMP-9 negative | MMP-9 positive | P | *r* |
| Tumor size |  |  |  |  |  |  |  |  |  |  |  |
| ≤2cm | 35 | 21 | 14 |  |  |  | 35 | 22 | 13 |  |  |
| >2cm | 16 | 3 | 13 | 0.006 | 0.358 |  | 20 | 15 | 5 | 0.356 | 0.124 |
| Histological grade |  |  |  |  |  |  |  |  |  |  |  |
| I/II | 35 | 16 | 19 |  |  |  | 36 | 22 | 14 |  |  |
| III | 16 | 8 | 8 | 0.776 | 0.040 |  | 19 | 15 | 4 | 0.180 | 0.178 |
| TNM |  |  |  |  |  |  |  |  |  |  |  |
| I | 22 | 17 | 5 |  |  |  | 24 | 16 | 8 |  |  |
| II/III | 29 | 7 | 22 | 0.000 | 0.466 |  | 31 | 21 | 10 | 0.933 | 0.011 |
| ER |  |  |  |  |  |  |  |  |  |  |  |
| Negative | 29 | 10 | 19 |  |  |  | 23 | 17 | 6 |  |  |
| Positive | 22 | 14 | 8 | 0.039 | 0.278 |  | 32 | 20 | 12 | 0.374 | 0.119 |
| PR |  |  |  |  |  |  |  |  |  |  |  |
| Negative | 27 | 9 | 18 |  |  |  | 21 | 17 | 4 |  |  |
| Positive | 24 | 15 | 9 | 0.037 | 0.280 |  | 34 | 20 | 14 | 0.089 | 0.223 |
| HER2 |  |  |  |  |  |  |  |  |  |  |  |
| Negative | 23 | 14 | 9 |  |  |  | 34 | 24 | 10 |  |  |
| Positive | 28 | 10 | 18 | 0.073 | 0.243 |  | 21 | 13 | 8 | 0.505 | 0.090 |
| Molecular subtype |  |  |  |  |  |  |  |  |  |  |  |
| Luminal A | 18 | 13 | 5 |  |  |  | 26 | 17 | 9 |  |  |
| Luminal B | 9 | 3 | 6 |  |  |  | 10 | 5 | 5 |  |  |
| HER2-positive | 19 | 7 | 12 |  |  |  | 11 | 8 | 3 |  |  |
| Triple-negative | 5 | 1 | 4 | 0.060^*^ | 0.358 |  | 8 | 7 | 1 | 0.426^*^ | 0.229 |

*Fisher's exact test

**Table B Univariate and multivariate analysis of overall survival in BCYW and BCMEW**

|  | BCYW | | | | |  | BCMEW | | | | |
| --- | --- | --- | --- | --- | --- | --- | --- | --- | --- | --- | --- |
|  | Univariate | |  | Multivariate | |  | Univariate | |  | Multivariate | |
|  | HR (95% CI) | P |  | HR (95% CI) | P |  | HR (95% CI) | P |  | HR (95% CI) | P |
| Tumor size |  |  |  |  |  |  |  |  |  |  |  |
| ≤2cm | 1.000 |  |  |  |  |  | 1.000 |  |  |  |  |
| >2cm | 3.822 (1.418-10.299) | 0.008 |  |  |  |  | 2.977 (0.711-12.469) | 0.136 |  |  |  |
| Histological grade |  |  |  |  |  |  |  |  |  |  |  |
| I/II | 1.000 |  |  |  |  |  | 1.000 |  |  | 1.000 |  |
| III | 1.377 (0.500-3.791) | 0.536 |  |  |  |  | 16.580 (2.035-135.113) | 0.009 |  | 14.319 (1.738-117.983) | 0.013 |
| ER |  |  |  |  |  |  |  |  |  |  |  |
| Negative | 1.000 |  |  |  |  |  | 1.000 |  |  |  |  |
| Positive | 0.509 (0.177-1.467) | 0.211 |  |  |  |  | 0.220 (0.044-1.090) | 0.064 |  |  |  |
| PR |  |  |  |  |  |  |  |  |  |  |  |
| Negative | 1.000 |  |  |  |  |  | 1.000 |  |  |  |  |
| Positive | 0.602 (0.218-1.657) | 0.326 |  |  |  |  | 0.339 (0.081-1.422) | 0.139 |  |  |  |
| HER2 |  |  |  |  |  |  |  |  |  |  |  |
| Negative | 1.000 |  |  |  |  |  | 1.000 |  |  |  |  |
| Positive | 1.555 (0.565-4.283) | 0.393 |  |  |  |  | 5.602 (1.129-27.783) | 0.035 |  |  |  |
| Molecular subtype |  |  |  |  |  |  |  |  |  |  |  |
| Luminal A | 1.000 |  |  |  |  |  | 1.000 |  |  |  |  |
| Luminal B | 3.434(0.766-15.393) | 0.107 |  |  |  |  | 2.894(0.407-20.558) | 0.288 |  |  |  |
| HER2-positive | 2.244(0.561-8.983) | 0.253 |  |  |  |  | 2.603(0.366-18.498) | 0.339 |  |  |  |
| Triple-negative | 5.102(1.027-25.347) | 0.046 |  |  |  |  | 3.496(0.492-24.833) | 0.211 |  |  |  |
| LNM |  |  |  |  |  |  |  |  |  |  |  |
| No | 1.000 |  |  | 1.000 |  |  | 1.000 |  |  | 1.000 |  |
| Yes | 8.531 (1.934-37.639) | 0.005 |  | 5.282 (1.156-24.132) | 0.032 |  | 19.458 (2.386-158.670) | 0.006 |  | 16.986 (2.060-140.065) | 0.009 |
| TNM staging |  |  |  |  |  |  |  |  |  |  |  |
| I | 1.000 |  |  |  |  |  | 1.000 |  |  |  |  |
| II/III | 7.050 (1.599-31.084) | 0.010 |  |  |  |  | 5.887 (0.724-47.860) | 0.097 |  |  |  |
| LMVD |  |  |  |  |  |  |  |  |  |  |  |
| Low | 1.000 |  |  |  |  |  | 1.000 |  |  |  |  |
| High | 1.207 (0.438-3.321) | 0.716 |  |  |  |  | 0.408 (0.097-1.712) | 0.221 |  |  |  |
| MMP-9 |  |  |  |  |  |  |  |  |  |  |  |
| Low | 1.000 |  |  | 1.000 |  |  | 1.000 |  |  |  |  |
| High | 8.610 (1.951-38.000) | 0.004 |  | 5.354 (1.171-24.481) | 0.031 |  | 1.313 (0.314-5.494) | 0.710 |  |  |  |
| VEGF-C |  |  |  |  |  |  |  |  |  |  |  |
| Low | 1.000 |  |  |  |  |  | 1.000 |  |  |  |  |
| high | 0.706 (0.265-1.883) | 0.487 |  |  |  |  | 0.550 (0.138-2.201) | 0.398 |  |  |  |

**Table C Univariate and multivariate analysis of disease-free survival in BCYW and BCMEW**

|  | BCYW | | | | |  | BCMEW | | | | |
| --- | --- | --- | --- | --- | --- | --- | --- | --- | --- | --- | --- |
|  | Univariate | |  | Multivariate | |  | Univariate | |  | Multivariate | |
|  | HR (95% CI) | P |  | HR (95% CI) | P |  | HR (95% CI) | P |  | HR (95% CI) | P |
| Tumor size |  |  |  |  |  |  |  |  |  |  |  |
| ≤2cm | 1.000 |  |  |  |  |  | 1.000 |  |  |  |  |
| >2cm | 2.835 (1.124-7.154) | 0.027 |  |  |  |  | 3.725 (0.931-140913) | 0.063 |  |  |  |
| Histological grade |  |  |  |  |  |  |  |  |  |  |  |
| I/II | 1.000 |  |  |  |  |  | 1.000 |  |  | 1.000 |  |
| III | 1.456 (0.564-3.760) | 0.437 |  |  |  |  | 8.158 (1.692-39.345) | 0.009 |  | 7.216 (1.482-35.140) | 0.014 |
| ER |  |  |  |  |  |  |  |  |  |  |  |
| Negative | 1.000 |  |  |  |  |  | 1.000 |  |  |  |  |
| Positive | 0.582 (0.218-1.551) | 0.279 |  |  |  |  | 0.183 (0.038-0.882) | 0.034 |  |  |  |
| PR |  |  |  |  |  |  |  |  |  |  |  |
| Negative | 1.000 |  |  |  |  |  | 1.000 |  |  |  |  |
| Positive | 0.835 (0.329-2.117) | 0.704 |  |  |  |  | 0.291 (0.073-1.162) | 0.081 |  |  |  |
| HER2 |  |  |  |  |  |  |  |  |  |  |  |
| Negative | 1.000 |  |  |  |  |  | 1.000 |  |  |  |  |
| Positive | 1.162 (0.458-2.946) | 0.752 |  |  |  |  | 6.456 (1.340-31.104) | 0.020 |  |  |  |
| Molecular subtype |  |  |  |  |  |  |  |  |  |  |  |
| Luminal A | 1.000 |  |  |  |  |  | 1.000 |  |  |  |  |
| Luminal B | 2.017(0.541-7.521) | 0.296 |  |  |  |  | 2.784(0.392-19.770) | 0.306 |  |  |  |
| HER2-positive | 1.301(0.397-4.268) | 0.664 |  |  |  |  | 3.891(0.650-23.295) | 0.650 |  |  |  |
| Triple-negative | 2.651(0.633-11.109) | 0.182 |  |  |  |  | 3.414(0.481-24.233) | 0.481 |  |  |  |
| LNM |  |  |  |  |  |  |  |  |  |  |  |
| No | 1.000 |  |  | 1.000 |  |  | 1.000 |  |  | 1.000 |  |
| Yes | 10.269 (2.353-44.821) | 0.002 |  | 6.466 (1.443-28.968) | 0.015 |  | 23.454 (2.925-188.050) | 0.003 |  | 21.549 (2.672-173.755) | 0.004 |
| TNM staging |  |  |  |  |  |  |  |  |  |  |  |
| I | 1.000 |  |  |  |  |  | 1.000 |  |  |  |  |
| II/III | 8.424 (1.932-36.738) | 0.005 |  |  |  |  | 7.026 (0.878-56.208) | 0.066 |  |  |  |
| LMVD |  |  |  |  |  |  |  |  |  |  |  |
| Low | 1.000 |  |  |  |  |  | 1.000 |  |  |  |  |
| High | 1.136 (0.440-2.932) | 0.792 |  |  |  |  | 0.346 (0.087-1.384) | 0.134 |  |  |  |
| MMP-9 |  |  |  |  |  |  |  |  |  |  |  |
| Low | 1.000 |  |  | 1.000 |  |  | 1.000 |  |  |  |  |
| High | 10.269 (2.353-44.821) | 0.002 |  | 6.466 (1.443-28.968) | 0.015 |  | 1.117 (0.279-4.466) | 0.087 |  |  |  |
| VEGF-C |  |  |  |  |  |  |  |  |  |  |  |
| Low | 1.000 |  |  |  |  |  | 1.000 |  |  |  |  |
| high | 0.713 (0.283-1.798) | 0.474 |  |  |  |  | 0.444 (0.119-1.655) | 0.227 |  |  |  |
